# Rhythm and Attention: Does the Beat Position of a Visual or Auditory Regular Pulse Modulate T2 Detection in the Attentional Blink?

**DOI:** 10.3389/fpsyg.2015.01847

**Published:** 2015-12-01

**Authors:** Christina Bermeitinger, Christian Frings

**Affiliations:** ^1^Experimental Psychology, Institute of Psychology, University of HildesheimHildesheim, Germany; ^2^Experimental Psychology, Department of Psychology, University of TrierTrier, Germany

**Keywords:** attentional blink, temporal attention, rhythm, pulse, alerting signals, audition and vision, multisensory processing

## Abstract

The attentional blink (AB) is one impressive demonstration of limited attentional capacities in time: a second target (T2) is often missed when it should be detected within 200–600 ms after a first target. According to the dynamic attending theory, attention cycles oscillatory. Regular rhythms (i.e., pulses) should evoke expectations regarding the point of the next occurrence of a tone/element in the rhythm. At this point, more attentional resources should be provided. Thus, if rhythmic information can be used to optimize attentional release, we assume a modulation of the AB when an additional rhythm is given. We tested this idea in two experiments with a visual (Experiment 1) or an auditory (Experiment 2) rhythm. We found large AB effects. However, the rhythm did not modulate the AB. If the rhythm had an influence at all, then Experiment 2 showed that an auditory rhythm (or stimulus) falling on T2 might generally boost visual processing, irrespective of attentional resources as indexed by the AB paradigm. Our experiments suggest that oscillatory cycling attention does not affect temporal selection as tapped in the AB paradigm.

## Introduction

A fundamental function of attention is the ability to select information in space or time given the limited capacities of the cognitive system. One impressive demonstration of limited attentional capacities in time is the attentional blink (AB) (for reviews see e.g., Shapiro et al., 1997; Dux and Marois, 2009; Martens and Wyble, 2010): within a rapid stream of irrelevant stimuli, a second relevant stimulus (target 2, T2) is often missed when it should be detected within 200–600 ms after a first relevant stimulus (target 1, T1; see below for more details). To optimize attentional precision, it would be beneficial if resources are provided at the right time. To determine when attentional resources should be provided, it might be helpful to use additional information such as previous knowledge, cues, primes, or context stimuli. In general, it is assumed that the incoming stream of events is partitioned by help of anticipated as well as actually presented stimuli (e.g., Klauer and Dittrich, 2010) to optimize the distribution of processing and response resources.

According to Barnes, Jones and colleagues (e.g., Large and Jones, 1999; Barnes and Jones, 2000; Jones et al., 2002), attention cycles oscillatory (see also e.g., Klimesch, 2012) when a rhythm is given. When the cognitive system is adapted to a given (auditory) regular rhythm (i.e., a pulse), the largest “attentional energy” is provided at that point in time at which the next rhythmic stimulus (i.e., a beat given by a tone) is expected. In other words, the rhythm is used to optimize the release of attentional resources. The experiments of Barnes and Jones (2000) investigated time perception. In several experiments, the authors presented isochronous auditory rhythms/pulses. The stimulus onset asynchrony (SOA) between two tones of the rhythm was always 600 ms. At the end of the rhythm, a standard interval was presented which was varied between 524 and 676 ms. Thereafter a comparison interval was presented, which was equally often shorter, equal to, or longer than the standard interval. Participants had to decide whether the comparison interval was shorter, equal to, or longer than the standard interval of the rhythm. Accuracy in categorizing the comparison interval was greatest for the expected SOA, that is, if the standard interval was exactly the same as the intervals in the preceding rhythm (600 ms). Accuracy was worst for very long or very short (i.e., very unexpected) standard intervals. The authors interpreted their results in the context of their dynamic attending theory.

Evidence for their theory of attentional deployment in time comes from neurophysiological studies in monkeys and humans as well as from behavioral studies (for reviews see e.g., Schroeder and Lakatos, 2009; Calderone et al., 2014). For example, in macaque monkeys, it was shown first, that neural oscillations modulate responses to stimuli and second, respond to external rhythmic stimuli. In the last case, intrinsic rhythms are entrained and shifted by extrinsic rhythms, resulting in optimization of neural responses when task-relevant events are expected (e.g., Lakatos et al., 2008). Predictable rhythmic beats are more easily perceived and faster detected than unpredictable (non-rhythmic) stimuli (e.g., Rohenkohl et al., 2012). Further, selective attention seems also closely related to entrainment to rhythms (Calderone et al., 2014). There are some recent studies showing that rhythm can drive the temporal allocation of attention and that orienting of attention is not modality dependent but even cross-modal (for uni-modal evidence see, for example, Doherty et al., 2005; Sanabria et al., 2011).

First, Bolger et al. (2013) used simple auditory and visual detection and discrimination tasks. They introduced a rhythm sequence (either with simple isochronous meter or with complex musical stimuli) prior to the occurrence of the stimuli which had to be detected. Reaction times depended on the metrical positions at which the stimuli were presented. The authors interpreted their results as evidence that metrical entrainment can enhance stimulus processing. Second, Miller et al. (2013) also found cross-modal influences of an auditory rhythm on the temporal attentional allocation to visual stimuli. These authors used regular or irregular tone sequences either synchronous or asynchronous to visual targets. Results showed faster saccadic detection responses (Experiments 1, 2) and improved accuracy in a discrimination task (Experiment 3) to visual targets coinciding with a tone of a regular rhythm compared to asynchronous (i.e., tone preceded or followed the visual target) as well as irregular rhythms.

Previous studies in which the influence of rhythms on attention and perception was investigated, focused on simple reaction time tasks (and sometimes accuracy tasks) to target stimuli. That is, one central aspect of attention—its limited capacity which is thought to be changing over time depending on stimuli which had to be processed—is not sufficiently touched by previous research on entrainment and/or rhythmic influences on attention. As already mentioned above, the AB paradigm is a suitable tool to investigate limitations of attention. In the visual domain, the AB reflects a robust deficit to correctly detect a second target (T2) appearing approximately 200–600 ms after a correctly identified first target (T1; e.g., Raymond et al., 1992). As a paradigm, the AB is most often studied by use of rapid serial visual presentation (RSVP) of shortly presented (distracting) stimuli, most often (strings of) letters, and varying the lag or SOA between the first and the second target. Typically, the first target has to be identified and the second target has to be detected or both targets have to be identified. Several theories might explain the AB (for an integration see e.g., Hommel et al., 2006). Whereas, early theories suggested a perceptual locus of the phenomenon (Raymond et al., 1992), later theories explained the AB at later, postperceptual stages of processing (e.g., Vogel et al., 1998; Jolicoeur and Dell'Acqua, 2000). The core element of most theories on the AB is based on capacity limitations of short-term memory or working memory. It is supposed that there are problems transferring and consolidating new information into working memory as long as preceding information is not processed to a certain level and that these processes related to the working memory draw on attentional resources (Hommel et al., 2006). Most likely, several mechanisms work together to result in an AB (Chun and Potter, 2001).

There are two studies in which entrainment and the AB were related. First, it was found that alpha entrainment (without an additional external rhythm except the RSVP rhythm) is larger for trials in which T2 cannot be reported than for trials in which T2 can be reported (Zauner et al., 2012). The authors argue that for stimuli presented with a frequency of about 10 Hz (i.e., approximately like the alpha frequency) those processes that underlie the generation of the P1 of the visual event related potential in the EEG (and that are related to alpha) interfere with those processes that enable the encoding of stimuli, specifically of T2. Second, there is recent work by Ronconi et al. (2015) who studied the influence of an acoustic or visual rhythmic stream before the RSVP stream, but with the same frequency. The authors presented entraining stimuli before the RSVP stream either with a regular rhythm, that is with the same frequency as the RSVP stimuli, or with an irregular rhythm, that is with variable interstimulus intervals between the entraining stimuli. There results showed reduced AB effects with a regular (compared to an irregular) rhythm.

**Figure 1:**
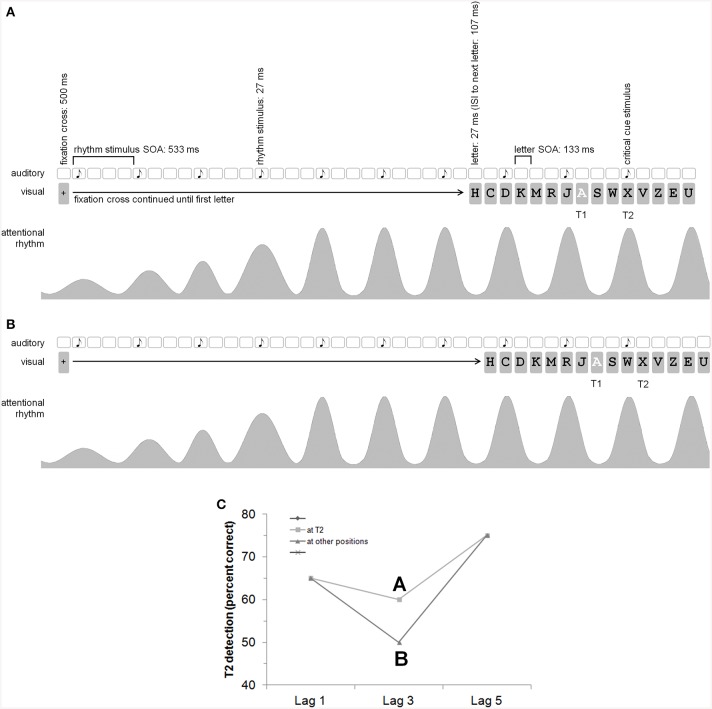
**General idea and procedure (with an auditory rhythm, i.e., Experiment 2)**. Please note that the time information is given with rounded values. With a refresh rate of 75 Hz, the exact timing is 26.66…, 106.66…, 133.33…506.66…, 533.33…**(A,B)** show the auditory rhythm presentation (the white squares indicate that there is no acoustic event at this time), the visual presentation (especially the RSVP with letters) with T2 at lag 3, as well as the attending/attentional rhythm. According to Barnes and Jones (2000), “an expected point in time corresponds to the peak of the attentional pulse carried by the oscillator” (p. 262). It is assumed that the oscillator adapts to stimulus time structure. **(A)** The auditory critical cue stimulus appears together with T2 which should result in a reduction of the AB (i.e., better T2 detection rates at lag 3; cf. **C**). **(B)** The auditory critical cue stimulus appears one position before T2. **(C)** Shown is our hypothesis for the AB effect depending on the rhythm and critical cue stimulus (which is either at T2 or at another position). The picture shows a reduction of the AB for T2s appearing together with the auditory critical cue stimulus (A better than B). We did not explicitly predict a general modulation of T2 detection by a rhythm—there might by a general enhancement or reduction of T2 detection also at lags 1 and 5. The main prediction, however, refers to the AB effect.

However, until now, there is now study in which the dependence of the AB effect on an additional rhythm like that used by Barnes and Jones (2000; see above) is studied. If other information, especially rhythmic information, can be used to optimize attentional release, we assume there should be a modulation of the AB when an additional rhythm is given. Specifically, we assume that the AB could be diminished by introducing a rhythm which peaks at the point in time when T2 is presented. In this case, the rhythm should evoke expectations regarding the point of T2 and more attentional resources should be provided at this point. All current theories concerning AB lead to the prediction that a peak of additional attentional resources corresponding to the onset of T2 should diminish the AB. That is, when a tone is expected at position T2, this should lead to a simultaneous release of attentional resources which in turn would lead to a diminished AB effect—given that a rhythm is able to release additional attentional resources. The general aim of the present experiments is to examine whether the assumed cyclical oscillating nature of attention in the presence of a rhythm can be manipulated to release attentional resources at peak times in the RSVP cycle, as would be shown by a reduction of the AB effect (for the general idea and procedure see also Figure 1).

In Experiment 1, we tested this prediction by using a visual rhythm before and during the RSVP stream. In Experiment 2, we used an auditory rhythm (Please note, in contrast to Ronconi et al., 2015, we did not investigate the question whether a regular or irregular rhythm—induced by entraining stimuli before the RSVP stream—enhances T2 performance. In their experiments and due to their research question, attention to each stimulus should be enhanced with a regular rhythm. In contrast, we tested the specific effect of rhythms falling at T2 vs. rhythms falling at stimuli surrounding T2).

## Experiment 1 (visual rhythm)

In Experiment 1, we used a visual rhythm (red symbols or letters) which was presented before the RSVP stream and continued during the RSVP stream. The last rhythm stimulus (= critical cue stimulus) appeared either one position before T2, at T2, one position after T2, or two positions after T2. Participants had no task regarding the rhythm. Their task was to indicate T1 identity and detect T2.

### Methods

#### Participants

The sample consisted of 29 students from Saarland University. Participants had normal or corrected-to-normal vision. They were paid for their participation or participated in exchange for course credit and gave informed written consent before participation. We excluded two participants as they made overall more than 60% errors in either the T1 or the T2 task. Of the remaining participants, 8 were male and 19 were female. Their median age was 24 years, ranging from 20 to 33 years.

The experiment was run in conformity with the ethical standards of our field and the AB task was approved by the ethical committee of the University of Hildesheim.

#### Design

Essentially, we used a 4 (position of the critical cue stimulus: one position before T2, at T2, one position after T2, two positions after T2) × 3 (lag: 1, 3, 5) design. Note that the factor Lag also determined the position of T1 (10, 8, 6) in the RSVP stream. Additionally, it was varied whether a T2 probe was presented or not. All factors were varied within participants. In the tradition of AB experiments, we used correct T2 probe detections when a T2 probe was presented (after correct T1 responses) as the dependent variable.

#### Material

##### Attentional blink task

The stimuli in the RSVP stream consisted of the letters of the alphabet except the letters I, O, and Q. Each letter except the X could appear at each position of the stream. X served exclusively as T2 probe letter and was presented in half of the trials. Stimuli were written in Courier New font (pt. 18, bold). Most of the letters were presented in black. T1 was presented in white. In Experiment 1, some letters were presented in red according to the rhythm. All letters were presented at the center of a gray background.

##### Critical cue stimulus and rhythm stimuli

The critical cue stimulus was embedded in a visually presented rhythm. For the visual rhythm, the critical cue stimulus, and the other rhythm stimuli were realized by colored letters or symbols. Before the RSVP stream began, participants saw rarely used symbols (e.g., ¥, Ø) written in red and with the same font and size as the letters in the RSVP stream. The symbols appeared in the same manner as the AB stimuli (i.e., at the center of the gray background; written in Courier New font, pt. 18, bold) and one after the other, to realize the rhythm. Overall, we used 14 different symbols. Seven randomly chosen symbols were presented in each trial. With the beginning of the RSVP stream, the rhythm continued by coloring the respective letters of the RSVP stream in red (or in white in the cases in which the rhythm coincides with T1). When the rhythm appeared together with T2, T2 was colored in red.

#### Procedure

Participants were individually tested in sound-attenuated chambers. The experiment was run using E-Prime software (version 1.3) with standard PCs connected to 17″ CRT monitors with a refresh rate of 75 Hz and standard QWERTZ-keyboards. Stimulus presentation was synchronized with the vertical retrace signal of the monitor. Viewing distance was about 60 cm. Instructions were given on the CRT screen. Participants had two tasks which were to be answered after each RSVP stream. First, participants answered the question (T1 identification): Which one was the white letter? They used the standard keyboard and entered the corresponding key. Second, participants answered the question (T2 detection): Was there an X after the white letter? Participants pressed the M-key (marked with JA = yes) or the C-key (marked with NEIN = no).

The sequence of each trial was as follows (see Figure 1 for an auditory variant): Participants started each trial self-paced by pressing the space-key. Then, a fixation stimulus (+) appeared at the center of the screen for 506.66…ms. Next, the first rhythm stimulus appeared for 26.66…ms. With a SOA of 533.33…ms, the next rhythm stimulus appeared. In each trial, seven rhythm stimuli were presented before the RSVP stream. After the seventh rhythm stimulus, there was an interval of 106.66…(critical cue stimulus one position after T2), 240 (critical cue stimulus at T2), 373.33…(critical cue stimulus one position before T2), or 506.66…(critical cue stimulus two positions before = after T2) ms. Then, the first letter of the RSVP stream appeared for 26.6…ms, followed by a blank screen for 106.66…ms. Thereafter, the next letter appeared (letter-to-letter SOA = 133.33…ms). Each RSVP stream contained 15 letters. The rhythm was continued during the RSVP stream with an SOA of 533.33…ms between two successive rhythm stimuli until the critical cue stimulus. The rhythm stimulus appeared simultaneously with a letter of the RSVP stream. There were three letters between two succeeding rhythm stimuli. T2 was always presented at position 11 of the RSVP stream. T1 was presented at position 10 (lag 1), 8 (lag 3), or 6 (lag 5) of the stream. There were 9, 7, or 5 distractor letters before T1, respectively, and 4 distractor letters after T2.

Each participant worked through five experimental blocks with 48 trials each. There was a short pause after each block. Before the first experimental block, there was a practice phase with 14 trials. Each experimental block consisted of 16 trials in which T2 was at lag 1 (i.e., directly after T1), 16 trials in which T2 was at lag 3, and 16 trials in which T2 was at lag 5. At position T2, half of the trials contained an X and the other half of the trials did not contain an X. Additionally, the critical cue stimulus appeared equally often one position before T2, at T2, one position after T2, and two positions after T2 in each lag (1, 3, 5) × T2 probe present (yes/no) condition. Within each block, conditions were presented in random order. Participants' task was to indicate first, which letter the white letter was and second, whether there was an X after the white letter or not.

### Results

Mean error rates were 11.9% (*SD* = 10.1) in the T1 task and 25.3% (*SD* = 12.2) in the T2 task. We first excluded trials with incorrect T1 responses. For the remaining trials (for each lag × position of critical cue stimulus there were between *M* = 8.3 and *M* = 9.3 observations after removal of trials with inaccurate T1 responses), we calculated mean correct T2 probe detections (in percent) when a T2 probe was presented. These mean correct T2 probe detections were subjected to a 4 (position of the critical cue stimulus) × 3 (lag) repeated measures ANOVA. The main effect of lag was significant, *F*_(2, 52)_ = 9.60, *MSE* = 1073.05, *p* < 0.001, $\eta_{\text{p}}^{2}=$ 0.27. This main effect reflected the AB: Repeated contrasts showed that there was a significant difference in correct T2 detections between lag 5 and lag 3, *F*_(1, 26)_ = 14.07, *p* = 0.001, but no difference between lag 3 and lag 1, *F*_(1, 26)_ < 1, *p* > 0.44.

**Figure 2:**
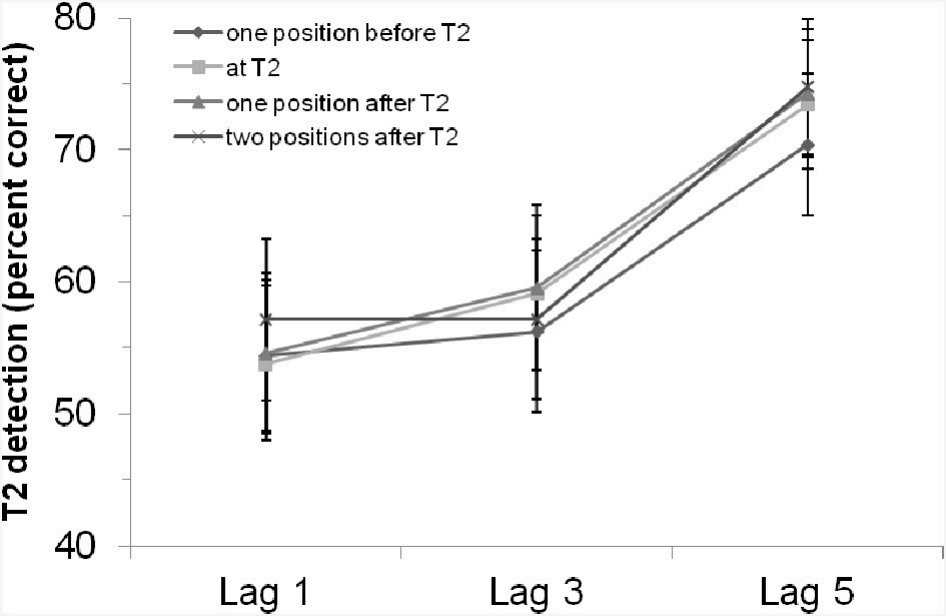
**T2 detection rate (in %, for trials with correct T1 identification and when the T2 probe was present) in Experiment 1 with a visual rhythm, depending on lag and position of the critical cue stimulus**. Bars indicate the standard error of the mean.

Neither the main effect of “position of critical cue stimulus,” *F* < 1, *p* > 0.65, nor the interaction effect, *F* < 1, *p* > 0.94, were significant. That is, there was no evidence for an influence of the rhythm on the general T2 detection rate or the AB. As shown in Figure 2, there was no better (but also no worse) T2 detection performance if the critical cue stimulus appeared simultaneously with T2.

As we only used trials with correct T1 performance for further analysis, of course, T1 performance in these trials was the same across conditions.

### Discussion

Using rhythmically and repeatedly presented colored visual stimuli before and during an RSVP—in which a critical stimulus could appear either at the position of T2, one position before T2, one position after T2 or two positions after T2—we found a significant visual AB with better detection rates at lag 5 than lag 3 (or lag1). However, there were no significant influences of the rhythm, neither in general nor in interaction with the AB. That is, the visual rhythm did not induce specific expectations or act as a general alerting signal. However, our results also show that the position of the critical cue stimulus does not *hamper* T2 detection, as there were no differences between the different positions of the critical cue stimulus.

## Experiment 2 (auditory rhythm)

In Experiment 2, we attempted to make the rhythm more salient/relevant and to approximate the rhythm to that of the experiments by Barnes and Jones (2000). Therefore, we used an auditory instead of a visual rhythm and added a task regarding the rhythm, to ensure that participants could not fully ignore the rhythm.

### Methods

#### Participants

The sample consisted of 43 students (9 male) from Saarland University with a median age of 22 years (ranging from 19 to 28). Participants had normal or corrected-to-normal vision. They were paid for their participation or participated in exchange for course credit and gave informed written consent before participation.

The experiment was run in conformity with the ethical standards of our field and the AB task was approved by the ethical committee of the University of Hildesheim.

#### Design, material, and procedure

The experiment was equal to Experiment 1 with the following exceptions. First, the rhythm was now realized auditorily with 1000 Hz tones presented via headphones for 27 ms each. The fixation cross remained on the screen until the onset of the first letter of the RSVP stream. Second, at the end of each trial and after the T1 and T2 response, participants indicated whether the rhythm was regular or not (note that each rhythm was actually regular); again, the answer was given by the M- or C-key. For this task, participants worked through a second practice phase directly after the first practice phase (with T1/T2 task) in which they practiced all three tasks (T1/T2/rhythm task). Third, each participant worked through five experimental blocks with only 24 trials each. Each block consisted of 8 trials in which T2 was at lag 1 (i.e., directly after T1), 8 trials in which T2 was at lag 3, and 8 trials in which T2 was at lag 5.

### Results

Mean error rates were 22.3% (*SD* = 11.4) in the T1 task and 30.0% (*SD* = 11.0) in the T2 task. Again, we first excluded trials with incorrect T1 responses. For the remaining trials (for each lag × position of critical cue stimulus there were between *M* = 3.7 and *M* = 4.0 observations after removal of trials with inaccurate T1 responses), we calculated mean correct T2 probe detections (in percent) when a T2 probe was presented. These mean correct T2 probe detections were subjected to a 4 (position of the critical cue stimulus) × 3 (lag) repeated measures ANOVA. If necessary, the Greenhouse-Geisser correction was applied, and corrected values are reported. The main effect of lag was significant, *F*_(1.48, 61.94)_ = 5.27, *MSE* = 2805.62, *p* = 0.01, $\eta_{\text{p}}^{2}=$ 0.11. This main effect reflected the AB: Repeated contrasts showed that there was a significant difference in correct T2 detections between lag 5 and lag 3, *F*_(1, 42)_ = 22.08, *p* < 0.001, but no significant difference between lag 3 and lag 1, *F*_(1, 42)_ = 1.88, *p* = 0.18.

**Figure 3:**
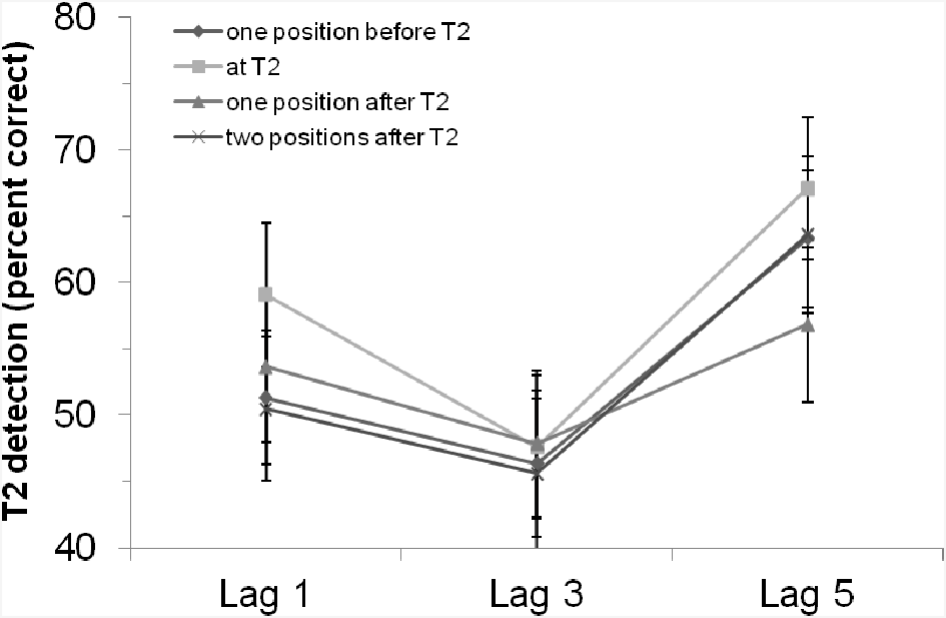
**T2 detection rate (in %, for trials with correct T1 identification and when the T2 probe was present) in Experiment 2 with an auditory rhythm, depending on lag and position of the critical cue stimulus**. Bars indicate the standard error of the mean.

The main effect of “position of critical cue stimulus” was not significant, *F*_(3, 126)_ = 1.64, *p* = 0.18. However, the planned contrast showed that T2 detection was marginally better if the critical cue stimulus appeared at T2 position compared to the other positions, *F*_(1, 42)_ = 3.77, *p* = 0.059. This revealed a tendency for enhanced attention when the critical cue stimulus appeared at T2. The interaction effect was not significant, *F* < 1, *p* > 0.54. That is, the rhythm had—if at all—a general effect on T2 detection, but was not able to modulate the AB. Figure 3 clearly shows that, especially at lag 3, there was no difference between the positions at which the critical cue stimulus appeared.

### Discussion

By use of an auditory rhythm before the critical auditory stimulus (again either coinciding with T2, or preceding or following T2), we again found a significant AB. Although the main effect of “position of critical cue stimulus” was again not significant, planned contrast revealed slight evidence for enhanced attention when the critical cue stimulus appeared at the point in time at which T2 was presented (compared to the other possible positions of the critical cue stimulus). Most interesting seems to be that there was no difference between the positions at which the critical cue stimulus appeared at lag 3 (which is the position with the largest AB). That is, the AB was again not modulated by the rhythm; if at all, the rhythm and the critical cue stimulus improved T2 detection irrespective of lag. This result might be interpreted as visual boosting due to an auditory stimulus. For example, better detection rates of visual stimuli were found with simultaneous presentation of an irrelevant auditory accessory stimulus (Frassinetti et al., 2002). Chen and Yeh (2009) could reduce or even reverse repetition blindness in a visual RSVP stream by presenting an auditory stimulus together with the stimuli of interest. We hasten to add that we created a cross-modal situation by using a visual AB task and an auditory rhythm. Perhaps, this might be a crucial difference to the experiments by Barnes and Jones (2000). However, when comparing the results of Experiment 1 (only visual) and 2 (visual and auditory), there were no large differences (see also below).

## General discussion

We analyzed the possible influence of oscillatory cycling attention on the AB. In particular, following Jones and colleagues (e.g., Barnes and Jones, 2000), we presented visual and auditory rhythms in a typical AB task. If attention adapts to the presented rhythm, the AB should depend on whether T2 is presented at a point in time when the attentional resources are at a maximum (due to the rhythm). However, although we found clear and large AB effects, we found not even the slightest hint of modulation of the AB effect by rhythm. If the rhythm had an influence at all, then Experiment 2 showed that an auditory rhythm (or stimulus) might generally boost visual processing at this particular point in time—irrespective of attentional resources as indexed by the AB paradigm.

Thus, the idea of oscillatory cycling attention as a model for the allocation of attentional resources in temporal selection (like in the AB task) does not hold. Participants obviously did not “use” (which is not necessarily meant in the controlled and/or conscious sense) the rhythm as a cue for increasing the allocation of attention although our rhythms were always perfectly reliable. In addition, note that we used two different variations of presenting the rhythm (visual and auditory) and also followed the procedures used by Jones and colleagues. This is important, because one may argue that it matters whether the rhythm is presented in the same modality as the to be attended stimuli (see Arend et al., 2006, who also concluded that the same AB attenuation effects resulted when additional stimuli were presented in the same or in another modality than the AB stimuli) or whether the modality in which the rhythm is presented “fits” to rhythm-processing in general (Welch et al., 1986)—of course it still might be the case that a particular combination of the modality in which the rhythm is presented and the modalities of rhythm and the RSVP stimuli might be a precondition for an effect of oscillatory cycling attention on the AB (e.g., maybe only rapid serial auditory streams are affected by auditory rhythms?). In addition, we must admit two possible caveats. First, we did not check in the same experiment whether our rhythm actually manipulated attention, but just failed to manipulate the AB (in other words, some kind of manipulation check concerning the effect of the rhythm would have been desirable). Second, the experiments conducted by Barnes, Jones, and colleagues (e.g., Large and Jones, 1999; Barnes and Jones, 2000; Jones et al., 2002) focused mainly on time perception or pitch judgments, which surely taps different attentional resources as compared to the AB. Thus, our data do not speak against these previous findings but only suggest that the model of oscillatory cycling attention is not easily applied to other tasks like the AB. It is clear that more research in different paradigms is needed to analyze whether the oscillatory cycling attention model could be applied to other domains than time perception and pitch judgments.

The fact that we observed—if any—generally slightly better T2 detection when an auditory stimulus coincided with the visual T2 fits to previous observations in RSVP streams which found visual boosting due to auditory stimuli (Frassinetti et al., 2002; Olivers and Van der Burg, 2008; Chen and Yeh, 2009). In particular, Olivers and Van der Burg (2008) found better T2 detection when an irrelevant bleep was presented together with T2 but not when it was presented directly before T2. This pattern suggests that the visual boosting is not due to alerting (because one might expect to find better detection performance if the auditory signal is presented shortly before T2) but due to multisensory enhancement. In fact, Busse et al. (2005) investigated whether neurophysiological signals to an irrelevant auditory stimulus were altered by a simultaneously presented, spatially (mis-)aligned visual stimulus. They found the strongest neurophysiological response to the irrelevant tone when the simultaneously presented visual stimulus was attended—suggesting some kind of multisensory enhancement of visual processing due to auditory stimulation (see Vroomen and de Gelder, 2000 for a discussion when auditory signals enhance or decrease visual processing).

There are a few papers in which a general enhancing effect of music/rhythm was found on T2 detection rates. Olivers and Nieuwenhuis (2005) found better T2 detection rates when participants listened simultaneously to a continuous rhythmic tune compared to the standard condition without music. The beat was not synchronized to the presentation of the stimuli in the RSVP stream. Better T2 detection rates were also found when participants should think about their holidays or their shopping plans for a dinner with friends simultanesously to the AB task. Also task irrelevant visual motion and flicker attenuates the AB (Arend et al., 2006). The authors suggested that a more diffuse attentional state causes better T2 detection rates, either via arousal or via positive affective state (see also Olivers and Nieuwenhuis, 2006). Ronconi et al. (2015) also found reduced AB effects when an auditory (but not when a visual) rhythm preceded the RSVP stream in the same frequency as the RSVP items. In general, however, there are also single reports, that the effect of music could not be replicated (Spalek and Di Lollo, unpublished data, as cited by Colzato et al., 2014). Further, differences between studies on entrainment and the AB (Zauner et al., 2012; Ronconi et al., 2015) used rhythms touching alpha. This also might explain differences in results. In this context, it also might be that the items of the RSVP stream themself induce a rhythm, too, which could generally enhance performance (in our experiments and all experiments using fixed time intervals between items in an RSVP stream).

**Figure 4:**
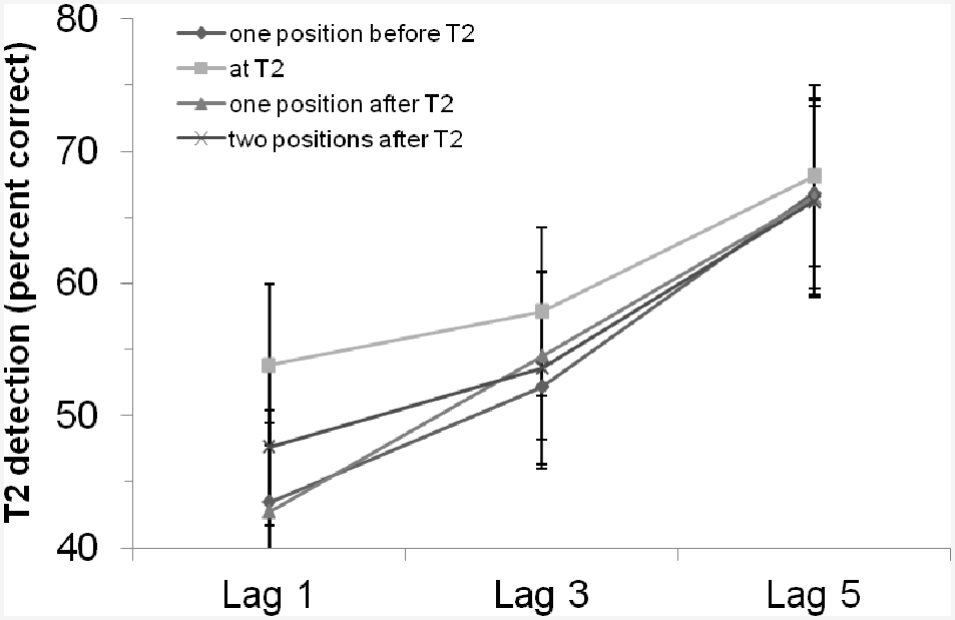
**T2 detection rate (in %, for trials with correct T1 identification and when the T2 probe was present) in the control experiment with an auditory critical cue stimulus without a preceding rhythm, depending on lag and position of the critical cue stimulus**. Bars indicate the standard error of the mean.

We ran a control experiment of Experiment 2 in which we removed the rhythm and presented only the critical cue stimulus. The experiment was a replication of Experiment 2 except that we did not present any rhythm but only single tones as critical cue stimuli. (Please find the detailed description of the control experiment in the Appendix in Supplementary Material.) The critical cue stimuli were tones between 750 and 1250 Hz and participants had to compare (same/different decision) the tone pitch of the critical cue stimulus with a 1000 Hz standard tone presented at the beginning of each trial. With 24 student participants, we again found a significant main effect of lag, i.e., an AB effect, *F*_(2, 46)_ = 9.30, *MSE* = 1050.33, *p* < 0.001, $\eta_{\text{p}}^{2}=$ 0.29. The main effect of “position of critical cue stimulus” as well as the interaction of both factors were not significant (*p*s > 0.40; for the results see also Figure 4). In addition, comparing the control experiment and Experiment 2, we did not find statistical evidence for a general enhancement or impairment by the rhythm (i.e., there was no main effect of experiment/rhythm, *F* < 1, *p* = 0.85), and the interaction of experiment/rhythm and lag also missed the criterion for being significant, *F*_(2, 128)_ = 1.99, *p* = 0.14 (all other effects including the factor experiment/rhythm were also not significant, *p*s > 0.70). Thus, we did not find evidence for a general enhancement/influence of the rhythm used in Experiment 2 and a control condition in which no rhythm was used (Of course, the lack of significance does not prove the H0). We interpret this as evidence that the results in our rhythm experiment(s) are not due a specific entrainment by the rhythm. As long as one does not argue that the presence of a critical cue stimulus effect and the rhythm modulation do interact in a disordinal way, the critical cue stimulus only adds a main effect and as a result the net effect of (any) critical cue stimulus effect and the rhythm modulation would still be usable for testing whether rhythms modulate the AB.

Why did we find no attenuation of the AB as it was found by Olivers and Nieuwenhuis (2005; 2006; but see Spalek and Di Lollo, unpublished data, as cited by Colzato et al., 2014) or Arend et al. (2006) when introducing a second task or enriching the material by further stimuli? One possible way (besides that some of the effects could not be replicated by Spalek and Di Lollo) to explain the difference between our experiments and that of Olivers and Nieuwenhuis or Arend et al. is that our additional task was not affectively positive (like shopping plans or music) and not as demanding like a flicker task. As a result, attentional resources were not allocated to the rhythm and thus the AB was not attenuated. In contrast to most of the other experiments, in which influences of rhythms/entrainment on perception and attention were found, we used an accuracy measure instead of reaction time measures. This difference might lead to differences in results. However, as Barnes and Jones (2000) also used accuracy measures, we should have found modulations of the AB effect.

Taken together, our experiments suggest that oscillatory cycling attention induced by the rhythms used does not affect temporal selection as tapped in the AB paradigm. Our results might also be interpreted as evidence that the tasks and materials used require different attentional networks with different oscillator frequencies (e.g., Fan et al., 2007; Posner, 2012). Future research could test whether regular and various kinds of irregular rhythms differ in their influence on the AB effect and whether longer/stronger entrainment phases lead to modulations of the AB—also in cases in which no beat is presented at T2 positions.

### Conflict of interest statement

The authors declare that the research was conducted in the absence of any commercial or financial relationships that could be construed as a potential conflict of interest.
