# Supplementary material for: Rhythm and Attention: Does the Beat Position of a Visual or Auditory Regular Pulse Modulate T2 Detection in the Attentional Blink?
Source: Front Psychol. 2015 Dec 1;6:1847. doi: 10.3389/fpsyg.2015.01847 (PMC4664834; doi:10.3389/fpsyg.2015.01847)
Supplement: Supplementary file 1 [file DataSheet1.PDF]

Appendix

X. Control experiment without rhythm

X.1 Method

X.1.1 Participants

The sample consisted of 24 students (15 male) from Saarland University with a median age of 21 years (ranging from 19 to 30). Participants had normal or corrected-to-normal vision. They were paid for their participation or participated in exchange for course credit and gave informed written consent before participation.

The experiment was run in conformity with the ethical standards of our field and the AB task was approved by the ethical committee of the University of Hildesheim.

X.1.2 Material and Procedure

The experiment was equal to Experiment 2 with the following exceptions. First, the rhythm was removed. Second, the critical cue stimulus was a tone between 750 and 1’250 Hz, in 50-Hz-steps. In half of the trials, the critical cue stimulus was 1’000 Hz, in the other half of trials, each 50-Hz-step was presented in 10% of the trials. The tone pitch of the critical cue stimulus was randomly selected for each trial. Third, to make the critical cue stimulus salient and to make the load comparable to Experiment 2, participants had the task to compare the tone pitch of the critical cue stimulus with a 1’000 Hz standard tone by indicating whether they had the same or different pitches. This standard tone was presented before each RSVP stream. The answer should be given after T1 and T2 response. The start of each trial was as follows: First, a fixation cross appeared for overall 1613.33… (critical cue stimulus one position after T2), 1746.66… (critical cue stimulus at T2), 1880 (critical cue stimulus one position before T2), or 2013.33… (critical cue stimulus two positions before T2) ms. With an SOA of 506.66… ms to the fixation cross, the standard tone was presented for 26.66… ms. After the fixation cross, the first letter of the RSVP stream appeared as in the previous experiments.

X.2 Results

Mean error rates were 17.7% (SD = 7.5) in the T1 task and 27.0% (SD = 11.4) in the T2 task. Mean correct T2 probe detections (in percent) when a T2 probe was presented (after correct T1 responses) were subjected to a 4 (position of the critical cue stimulus) x 3 (lag) repeated measures ANOVA. The main effect of lag was significant, $F(2, 46) = 9.30$, MSE = 1050.33, $p < .001$, $\eta_p^2 = .29$. Again, this main effect reflected the attentional blink: Repeated contrasts showed that there was a significant difference in correct T2 detections between lag 5 and lag 3, $F(1, 23) = 11.08$, $p = .003$, but no significant difference between lag 3 and lag 1, $F(1, 23) = 2.64$, $p = .12$.

The main effect of “position of critical cue stimulus” was not significant, $F(3, 69) < 1$, p = .40. Especially, the difference between the position of the critical cue stimulus at T2 and the other positions missed the criterion for being significant (planned contrast: $F(1, 23) = 2.59$, p = .12). The interaction effect of the factors “position of critical cue stimulus” and lag was not significant, $F < 1$, $p > .90$.
